# Two Cases of Penetrating Bayonet Wounds of the Chest

**Published:** 1864-09

**Authors:** S. Baruch

**Affiliations:** 3d South Carolina Battalion, Kershaw's Division.


ART. III.—*Two Cases of Penetrating Bayonet Wounds of the Chest.* By Surgeon S. BARUCH, 3d South Carolina Battalion, Kershaw's Division.

Case 1.—Private Wm. Tinkler, company "G.," 3d battalion S. C. infantry, was wounded in a bayonet charge of the enemy on the 8th May, at Spottsylvania Court House, Virginia. He was brought to the infirmary an hour after reception of his wound. On examination, two apertures were discovered, one a triangular, ragged opening in the back, about one-half inch to the right of the tenth dorsal vertebra, and the other a small puncture, three inches below the right nipple, near the angle of the ninth rib.

He states that as the enemy's line advanced he was in a stooping posture, loading and firing rapidly. While thus engaged he was transfixed by a bayonet, and he asserts positively that he distinctly felt the withdrawal of the weapon.

When first brought to me, the following symptoms presented themselves. His face was pallid and anxious, nostrils distended; skin cool; pulse weak, but somewhat excited; breathing difficult and labored. There was slight oozing of blood from the posterior orifice, *which was contracted*, and bloody expectoration similar to that occurring in gunshot injuries of the lungs. These symptoms, conjoined with the direction of the wound, led me to the belief that the right lung was transfixed. The "bent" position of the patient prevented implication of the right lobe of the liver, as in that posture the liver is separated from the diaphragm, and thus the bayonet avoided this organ.

May 9th.—Patient expectorates bloody mucus, complains of pain in the right lung, has but little cough. Shock has passed off and he is tranquil. May 12th. Bloody expectoration ceased, but pain still continues. Auscultation and percussion reveal no signs of pneumonia or pleuritis. May 13th. Posterior wound is healed over by scabbing. Still no symptoms of pneumonia. May 16th. Patient is doing finely; there is some acceleration of the circulation and dyspnœa, but no physical symptoms of lung disease. May 17th. Sent to General Hospital in fine spirits.

Case 2.—Corporal G. Percival, company "F.," 3d South Carolina battalion, a man of large frame and good constitution, was wounded in a desperate charge on Kershaw's brigade, on the 8th May.

His case was brought to my notice about three hours after infliction of the wound.

Divesting him of his shirt, which had an opening in the back, I examined the chest carefully, and found two apertures of a triangular shape, one, the largest, in the back, just below the inferior angle of the left scapula, and the other, very small, over the sternum, near its right border, on a line with the fourth rib.

Patient was lying on his abdomen and partially on his left side, behind a small rail-pile when he was transfixed by a bayonet. When brought to the infirmary his countenance was pale, and *did not* wear that expression of anxiety so peculiar in penetrating wounds of the chest; his symptoms indicated a shock to the nervous system, induced by the intense excitement of a hand-to-hand conflict with a drunken and infuriated foe. The whole system seemed to suffer from depression following great excitement. Acting on this supposition, I administered some stimulants and anodynes, which partially restored the patient, and enabled him to recite his encounter with the enemy.

Under this treatment the pulse rallied and skin became warm. There was but slight dyspnœa, no cough and but little bloody expectoration, indicating that the injury to the lung was not extensive. A careful investigation of the posture of the patient during the reception of the wound and a consideration of its direction convinced me that the weapon grazed the right border of the posterior portion of the left lung, passing through the posterior mediastinum and evading the heart, which was displaced by the patient lying somewhat on his left side. May 9th. Patient has recovered from the shock and nervous depression; this fact confirmed me in the belief that the lung was not seriously implicated, and the heart and great vessels escaped entirely. His countenance seems much more cheerful; pulse somewhat excited, but it is more of a nervous than febrile excitement. Very slight expectoration of bloody mucus, and he coughs only when he makes an effort to change his position. May 11th. Doing well, dyspnœa inconsiderable, and no physical signs of pneumonia or pleuritic inflammation. His bowels having been costive, a mild cathartic was ordered. May 16th. Still free of pulmonary inflammation, but he complains of pain in the epigastric and umbilical region. Emollient poultices were applied, and anodyne administered at bedtime. May 17th. Pain has discontinued, and patient is again in good spirits. May 19th. Sent to General Hospital.

Both patients are now—in July—on duty with their command.

*Remarks.*—Besides these cases, a number of others of a less serious nature presented themselves for treatment, nearly all of which recovered at the field infirmary and were returned to duty.

The limited experience derived from the treatment of these cases induces me to consider bayonet wounds as very simple injuries, readily healing by first intention.

Perfect tranquility and continued application of cold water are the most important remedial means. I found that the chest cases, though presenting a more extensive track than those of the hand, loins and shoulder, healed more rapidly, for this reason, that the slightly punctured patient would not obey my injunctions to keep quiet.

The *sequelæ* of bayonet wounds are not as serious as those of gun-shot injuries. Not one of the men who received an injury of this kind on the 8th May, has been rendered unfit for service. There is no exudation of superfluous lymph, no irregular gluing of muscular fibres, no permanent or even temporary contraction of muscles and tendons.

Our knowledge of bayonet wounds has been so limited that their effects have been, until a recent period, involved in considerable doubt and even mystery. Experience, however, teaches that we have exaggerated the nature of these injuries, and attributed to them formidable qualities which they happily do not possess.

Is it not our duty to convey the teachings of experience to the noble men who are daily confronting the foe, and to the amelioration of whose sufferings our labors are devoted?

Why is it that soldiers have such a wholesome fear of the bayonet? Why is it that the determined approach of a line of glistening steel makes the cheek blanch and causes the bravest hearts to waver? Why do we in every battle witness the rout of lines that have unflinchingly withstood a continued galling fire of musketry and artillery, as soon as the opposing line approaches closely with fixed bayonets?

This dread of "cold steel" is, in my humble opinion, mainly attributable to ignorance of the nature of the injuries inflicted by it. There appears to exist in the minds of men a vague dread of transfixion by the bayonet. But this would not be so were it generally known that bayonet wounds are almost harmless, when compared to the ploughed tracks which the terrible minie bores through the tissues. The bayonet is easily diverted from a straight course by bony, cartilaginous and tendinous tissues, and forms a smooth track, whilst the minie is relentless in its course, whirling with unimpeded force through all opposing structures, crushing, tearing, maiming all. A bayonet wound almost invariably heals by first intention under auspicious circumstances, and leaves no deformity behind, whilst the simplest ball wound requires weeks for a complete recovery, and then, perhaps, leaves the sufferer with a contracted and useless limb.

It is not my purpose to write an elaborate essay on these injuries. I merely desire to call the attention of my colleagues to the necessity of a more searching investigation of their nature, and to the advantages that would accrue were our soldiers made to appreciate the immense difference in the effects of bayonet and gun-shot injuries.

